# Novel aggrecan variant, p. Gln2364Pro, causes severe familial nonsyndromic adult short stature and poor growth hormone response in Chinese children

**DOI:** 10.1186/s12881-018-0591-z

**Published:** 2018-05-16

**Authors:** Dandan Xu, Chengjun Sun, Zeyi Zhou, Bingbing Wu, Lin Yang, Zhuo Chang, Miaoying Zhang, Li Xi, Ruoqian Cheng, Jinwen Ni, Feihong Luo

**Affiliations:** 1 Department of Pediatric Endocrinology and Inherited Metabolic Diseases, Children’s Hospital of Fudan University, 399 Wan Yuan Road, Minhang District, Shanghai, 201102 China; 2 College of Letters and Science, University of California, Berkeley, USA; 3 Pediatrics Research Institute, Children’s Hospital of Fudan University, Shanghai, China

**Keywords:** Aggrecan, *ACAN* gene, Short stature, Adult height, Growth hormone

## Abstract

**Background:**

Mutations in the aggrecan (*ACAN*) gene can cause short stature (with heterogeneous clinical phenotypes), impaired bone maturation, and large variations in response to growth hormone (GH) treatment. For such cases, long-term longitudinal therapy data from China are still scarce. We report that a previously unknown *ACAN* gene variant reduces adult height and we analyze the GH response in children from an affected large Chinese family.

**Methods:**

Two children initially diagnosed with idiopathic short stature (ISS) and a third mildly short child from a large Chinese family presented with poor GH response. Genetic etiology was identified by whole exome sequencing and confirmed via Sanger sequencing. Adult heights were analyzed, and the responses to GH treatment of the proband and two affected relatives are summarized and compared to other cases reported in the literature.

**Results:**

A novel *ACAN* gene variant c.7465 T > C (p. Gln2364Pro), predicted to be disease causing, was discovered in the children, without evident syndromic short stature; mild bone abnormity was present in these children, including cervical-vertebral clefts and apophyses in the upper and lower thoracic vertebrae. Among the variant carriers, the average adult male and female heights were reduced by − 5.2 and − 3.9 standard deviation scores (SDS), respectively. After GH treatment of the three children, first-year heights increased from 0.23 to 0.33 SDS (cases in the literature: − 0.5 to 0.8 SDS), and the average yearly height improvement was 0.0 to 0.26 SDS (cases in the literature: − 0.5 to 0.9 SDS).

**Conclusions:**

We report a novel pathogenic *ACAN* variant in a large Chinese family which can cause severe adult nonsyndromic short stature without evident family history of bone disease. The evaluated cases and the reports from the literature reveal a general trend of gradually diminishing yearly height growth (measured in SDS) over the course of GH treatment in variant-carrying children, highlighting the need to develop novel management regimens.

**Electronic supplementary material:**

The online version of this article (10.1186/s12881-018-0591-z) contains supplementary material, which is available to authorized users.

## Background

Human linear growth is a complex process determined primarily by genetic factors and modulated by environmental factors [1]. Based on its etiology, short stature can be divided into primary growth disorder, secondary growth disorder, and idiopathic short stature (ISS) [2, 3]. ISS refers to short stature with no apparent cause and is often described as either sporadic cases or familial short stature [2]. With the development of sequencing technologies, ISS has been found, in some patients, to be caused by mutations in genes involved in the hypothalamic-pituitary-growth hormone (GH) axis, such as *GH1, GHR,* and *GHRHR* (*GHRH* receptor) [4–7]. Genetic defects in the GH axis only constitute a small fraction of clinically diagnosed short-stature cases [8]. Defects in growth height are probably associated with other yet-to-be-identified genes [9].

Recent studies found that *ACAN* (MIM 155760, NM_013227.3) mutations are associated with accelerated bone maturation and progressive growth failure [4, 10]. In a prospective clinical study of 290 patients born small for gestational age (SGA) with short stature [11], four patients were identified as carrying heterozygous *ACAN* mutations and showed large variation in response to GH treatment. A recent study indicated that *ACAN* mutations are associated with short stature without syndromic manifestations [12]. However, studies on the relationship between *ACAN* gene mutations and adult height are scarce, and the reported data are typically from small family pedigrees. Thus, the optimal management of child mutation carriers remains unclear. We have analyzed the effects of an *ACAN* variant on adult height, based on data from one large Chinese family. In addition, we report the affected children’s response to GH treatment and summarize the effects of GH treatment on individuals with *ACAN* mutations reported in the literature.

## Methods

### Subjects and diagnosis

**Fig. 1:**
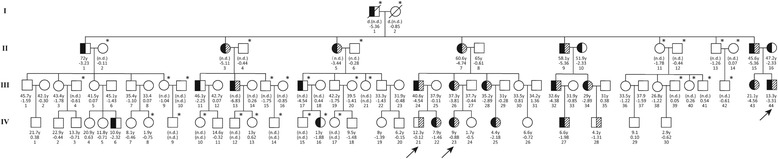
The family pedigree of members with and without the *ACAN* p. Gln2364Pro variant. Age and height are listed below each symbol. Black arrows: three children whose bloods were performed WES. Half-black symbols: family members with short stature (height < -2SD). Half-shaded symbols: family members carrying the *ACAN* mutation. An asterisk next to a symbol indicates that a blood sample was unavailable. A slash with a symbol indicates that the individual is deceased. n.d.: not determined

This study was approved by the Hospital Ethics Committee of the Children’s Hospital of Fudan University. Three children from the same large family, comprising 90 members, were recruited. Two of the children (Fig. 1. IV:23 and IV:44) were initially referred to us due to their short statures, according to the growth charts for Chinese children and adolescents aged 0 to 18 years [13]. Normal GH secretion in both children was confirmed by a peak of GH ≥10 ng/mL in insulin or arginine provocative tests. After evident physical deformities and organic etiologies were excluded, the primary diagnosis of ISS was made according to the diagnosis consensus [3]. The third child, for whom an initial height record was lacking, had height (Fig. 1. III:21) -0.88 standard deviation score (SDS) from the same family and had received 1 year of GH therapy before being referred to our clinic. We initiated GH therapy in these three children at a dose between 40 and 60 μg/kg/d and titrated to maintain serum IGF-1 levels between the average and + 2 standard deviations (SDs) of the reference [3]. All three children showed a poor response to GH treatment according to the criterion of SDS < 0.5 proposed by Patel and Clayton [14]. Therefore, the three children and their family members were offered genetic testing. Among the 90 family members, 62 subjects were available.

### Whole exome sequencing

Whole exome sequencing was performed with genomic DNA extracted (QIAamp DNA Blood Mini kit, Qiagen, Germany) from subjects’ blood samples. Exomes were enriched with Illumina’s SureSelect Human All Exon kit V4, targeting 50 Mb of sequence from exons and flanking regions. Sequencing was performed with the Illumina HiSeq 2000 platform. Reads with adaptors, reads with > 10% of unknown bases (Ns), and low-quality reads with > 50% of low-quality bases (i.e., bases with a sequencing quality of 5 or less) were discarded from the raw data to generate clean reads, 90-bp paired-end and with at least 100-fold average sequencing depth. Clean reads were aligned to the reference human genome (UCSC hg19) by using the Burrows-Wheeler Aligner (BWA) (v.0.5.9-r16). Subsequent processing steps of sorting, merging, and removing duplicates for the BAM files were performed by using SAMtools and Picard (http://broadinstitute.github.io/picard/). Mutation calls, which differed from the reference sequence, were obtained with the use of GATK. Mutations were annotated by ANNOVAR and VEP software [15, 16]. Mutations with suboptimal quality scores were removed from consideration. The remaining mutations were compared computationally with the list of reported mutations from the Human Gene Mutation Database (HGMD, professional version). Mutations in this database with minor allele frequency < 5% according to either the 1000 Genomes Project or ExAC data of The Exome Aggregation Consortium (http://exac.broadinstitute.org/) were retained. For changes that were not represented in the HGMD, synonymous mutations, intronic mutations that were > 15 bp from exon boundaries (which are unlikely to affect messenger RNA splicing), and common mutations (minor allele frequency > 1%) were discarded [15, 16].

### Bioinformatics assessment of variant function

To assess the effects of the variant, we conducted analyses using SIFT, PolyPhen-2, and MutationTaster software, to predict possible deleterious effects [17–19]. In addition, we performed conservation analysis, using the PhyloP score embedded in the USCS genome browser [20]. Three-dimensional models of aggrecan protein were produced by SWISS-MODEL SERVER [21].

### Variant confirmation by sanger sequencing

After the identification of an *ACAN* variant in family members, we performed Sanger sequencing on PCR products containing the variant (in exon 14). The purified DNA was used as template for PCR amplification. The reaction mixture (20 μL) contained 50 ng DNA; 1 U Taq DNA polymerase (Promega, USA); 1.5 mM MgCl_2_; 0.25 mM each dNTP; and 0.5 μM primers. The primers for the amplification of *ACAN* were as follows: *ACAN*-Forward: CATCTGCCATCCCCTGGT, *ACAN*-Reverse: CCTACACCGCCACTCTCCTC. The thermal cycling conditions for the PCR reactions consisted of an initial denaturation step at 95 °C for 5 min; 35 cycles of denaturation at 95 °C for 30 s, primer annealing at 58 °C for 30 s, and extension at 72 °C for 30 s; and a final step at 72 °C for 7 min. The PCR products were sequenced in an ABI sequencer 3500 xL GA (Applied Biosystems). The sequence data were evaluated using Mutation Surveyor software and compared with the reference sequence of *ACAN* (NM_001135).

### Family members

After identification of *ACAN* variant in the three children who came to our clinic, we invited all family members to participate in our study; the pedigree is shown in Fig. 1. Genotyping and collection of basic information (height, age, and history of bone disorders) in the adult family members were conducted after obtaining informed consent from the subjects. For persons younger than 18 y, informed consent was obtained from the parents.

### Measurement of responses to growth hormone treatment

During GH treatment of the three children, we measured the levels of serum IGF-1 and IGF-binding protein 3 (IGFBP3) every three or six months by solid-phase, two-site chemiluminescent immunometric assay via an automated MMULITE 1000 immunoassay system (Siemens, Munich, Germany).

### Statistical analysis

Height, body mass index (BMI, weight (kg) divided by the square of the height (m)), and bone age (GP atlas) were measured. SDS values for height and BMI were calculated using national references. We used a t-test (SPSS Version 17.0) to compare the differences in height between family members with and without the *ACAN* variant.

## Results

### ACAN variant and functional in silico prediction

**Fig. 2:**
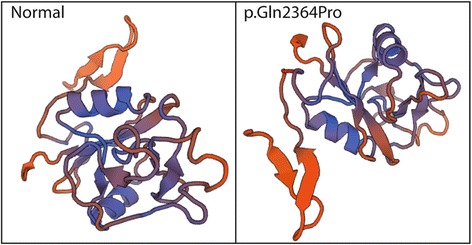
The three-dimensional models for aggrecan protein with and without the variant

Whole exome sequencing to a median of 150× 125.47 (125.47~ 165.65) coverage in the index patients identified 825,823 genetic variants of which 119 were not found in dbSNP137, ExAC, the 1000 Genomes database, or in internal database at 0.5% allele frequency. Further analysis showed that only one variant, c.7465 T > C (p. Gln2364Pro), was consistent with the phenotype of the index and shared by the three affected children in this family. Subsequent Sanger sequencing detected the same variant (p. Gln2364Pro) totally in 19 of 62 blood samples available which was not present in the HGMD or GNOMAD databases. These 19 subjects (10 females and 9 males) ranged in age from 4.1 to 60.6 y and included 7 children (< 18 y; 3 females, 4 males) and 12 adults (7 females, 5 males). The missense variant was predicted by in silico tools to be deleterious (Table 1). Amino acid conservation analysis showed that the affected site was highly conserved in at least fifteen species, including humans (Additional file 1: Figure S1). The three-dimensional models for aggrecan protein with the variant, produced by SWISS-MODEL SERVER (Fig. 2), showed significant anomalies in the formation of normal dimensional structure.

**Table 1:** Characteristics of children with *ACAN* variant treated with growth hormone

	Relative 1	Relative 2	Proband
Gender	Female	Male	Male
Birth weight (kg)	3	3	3.5
Birth height (cm)	50	51	50
*ACAN* mutation	c.7465 T > C^a^	c.7465 T > C^a^	c.7465 T > C^a^
Protein changes	p.Gln2364Pro	p.Gln2364Pro	p.Gln2364Pro
First visit			
Age (y)	5.6	7.8	6.4
Height (cm)	104.5	122.5	100.5
HSDS	− 2.09	− 0.88	− 3.74
Weight (kg)	18	25	17.5
BMI (kg/m^2^)	16.48	16.66	17.33
Peak growth hormone (ng/ml)	12.1	ND	15.1
IGF1(ng/ml)	290	200	148
IGFBP3 (μg/ml)	4.15	7.96	3.43
Bone age (y)	6.5	9.5	6

*HSDS* height standard deviation scores, *BMI* body mass index, *IGF*1 insulin-like growth factor 1, *IGFBP*3 insulin-like growth factor binding factor 3, *ND* not detected; ^a^ in silico *prediction*: Sift: affect protein function (score = 0.00); Polyphen: probably damaging (score = 1)

### ACAN variant and its correlation with adult height

Among the 31 adult family members (15 females, 16 males), there were 12 adult variant carriers (7 females, 5 males). All of the adults with the *ACAN* mutation had severe short stature (height < − 2 SD, Table 2), and the difference in height between members with and without the *ACAN* variant was significant in both genders (*p* < 0.001, Table 2).

**Table 2:** Adult heights of family members with and without *ACAN* variant

	Variant (+)	Variant (−)	*P* value
Male	(*n* = 5)	(*n* = 11)	
Height (cm)	141.2 ± 4.4	162.2 ± 8.1	< 0.0001
HSDS	−5.2 ± 0.7	−1.7 ± 1.3	
Female	(*n* = 7)	(*n* = 8)	
Height (cm)	139.4 ± 4.9	156.0 ± 5.8	< 0.0001
HSDS	−3.9 ± 0.9	− 0.9 ± 1.1	

Data are presented as mean ± SD. *HSDS* height standard deviation score

### ACAN variant and response to GH treatment

**Fig. 3:**
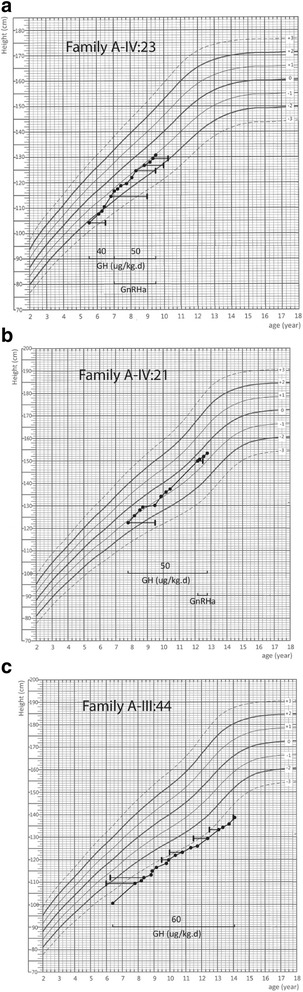
Growth charts of patients with the *ACAN* p. Gln2364Pro variant. Vertical bars represent bone age. GH; growth hormone. GnRHa; gonadotropin releasing hormone analog

The major characteristics of the three children who received GH treatment are shown in Table 1. The proband (Fig. 1, III:44) was a boy born with normal birth length and weight. However, his father’s height was 153 cm (− 5.36 SDS) and his mother’s height was 140 cm (− 2.33 SDS). He was referred to us at the age of 6.4 y with height 100.5 cm (− 3.74 SDS). After GH therapy, 60 μg/kg/d for 8 years, his height was − 3.75 SD below average, at latest evaluation (age 14.1 y, Fig. 3c).

The affected relative 1 in the family was a girl (Fig. 1. IV:23), born full term, with normal birth length and weight. She was referred to our clinic at the age of 5.6 y with height 104.5 cm (− 2.09 SDS) and bone age (BA) 6.5 y. Her father’s height was 170 cm (− 0.44 SDS), and her mother’s height was 138 cm (− 4.19 SDS). Radiographs showed slight abnormalities of her spine, including cervical-vertebral clefts and apophyses in the upper and lower thoracic vertebrae (Additional file 2: Figure S2). We started GH treatment at dose 40 μg/kg/d, and she received GnRHa for pubertal management at age 7. Her height was − 1.07 SDS at her latest visit (age 9.5 y, Fig. 3a).

Relative 2 (Fig. 1. IV:21) was a boy born with normal birth length and weight at full term. He visited our department at the age of 7.8 y with height 122.5 cm (− 0.88SDS). His father’s height was 150 cm (− 3.72 SDS), and his mother’s height was 160 cm (− 0.11 SDS). His bone age (BA) was 9.5 y at his first visit. He received GH therapy, 50 μg/kg/d (Fig. 3b). He received GnRHa at 12.2 y due to his pubertal development. His latest height was − 0.31 SD (at age 12.8 y, Fig. 3b).

### Responses to GH treatment reported in other studies

**Fig. 4:**
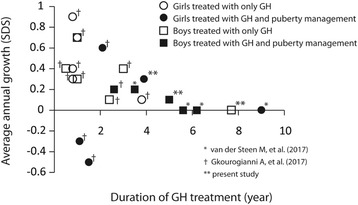
Average annual change in the SDS of height in subjects with *ACAN* p. Gln2364Pro variant receiving GH treatment

In addition to reporting on our current cases, we reviewed the two other available studies on children with an *ACAN* variant, which involved 18 patients. The characteristics of these patients and their responses to GH treatment are shown in Table 3. The change in height SDS during the first year of treatment ranged from − 0.5 SDS to 0.8 SDS, and the overall yearly height change during GH treatment was − 0.5 SDS to 0.9 SDS. Among these children, there was a general trend of a gradual reduction in yearly height SDS growth over the course of GH treatment (Fig. 4).

**Table 3:** Patients with *ACAN* variant treated with growth hormone

	N	Gender	*ACAN Variant*	GH starting age (y)	Initial Height (SDS)	GH dosage	1st year growth Δ_SDS_	GH duration (y)	Additional treatment	Average growth Δ_SDS_/y	Latest Height (SDS)
van der Steen M, et al. (2017)											
	1	Female	c.1608C > A (p.Tyr536*)	5.0	−3.7	1-2 mg/m2/d	+ 0.7	9	GnRHa for 1.5 y	−0.0	− 3.9 (AH)
	2	Male	c.1608C > A (p. Tyr536*)	11.9	−2.4	2 mg/m2/d	+ 0.7	3.5	GnRHa for 2 y	+ 0.2	−1.6
	3	Male	c.7090C > T (p.Gln2364*)	11.7	−2.7	2 mg/m2/d	+ 0.1	5.6	GnRHa for 2 y	−0.0	−2.9
	4	Male	c.4762_4765del (p.Gly1588fs)	12.3	−2.7	1 mg/m2/d	0	6.2	GnRHa for 2 y	+ 0.0	−2.6 (AH)
Gkourogianni A, et al. (2017)											
	1	Male	c.272delA (p.Arg93Alafs*)	8.7	−3.3	30-50 μg/kg/d	+ 0.8	2.6	Along with aromatase inhibitor	+ 0.2	−2.7
	2	Male	c.272delA (p.Arg93Alafs*)	6.3	−1.8	30-50 μg/kg/d	+ 0.2	2.4		+ 0.1	−1.6
	3	Female	c.7064 T > C (p.Leu2355Pro)	12.0	−3.2	30-50 μg/kg/d	−0.5	1.5	Along with GnRHa	−0.5	−3.9 (AH)
	4	Male	c.5391 (p.Gly1797Glyfs*)	6.2	−2.6	30-50 μg/kg/d	+ 0.6	1.0		+ 0.3	−2.0
	5	Female	c.7429G > A (p.Val2417Met)	11.3	−0.8	30-50 μg/kg/d	−0.2	1.1	Along with GnRHa	−0.3	−1.1
	6	Female	c.7429G > A (p.Val2417Met)	5.5	−1.2	30-50 μg/kg/d	+ 0.4	3.8		+ 0.1	−0.7
	7	Female	c.1443G > T (p.Glu415*)	8.5	−1.7	30-50 μg/kg/d	+ 0.6	2.1	GnRHa for 1 y	+ 0.6	−0.5
	8	Male	c.1443G > T (p.Glu415*)	5.7	−1.7	30-50 μg/kg/d	+ 0.2 (0.5y)	0.5		+ 0.4	−1.5
	9	Female	c.1443G > T (p.Glu415*)	8.4	−0.7	30-50 μg/kg/d	+ 0.2 (0.8y)	0.8		+ 0.3	−0.5
	10	Female	c.1443G > T (p.Glu415*)	3.2	−3.0	30-50 μg/kg/d	+ 0.7 (0.8y)	0.8		+ 0.9	−2.3
	11	Female	c.4657G > T (p.Glu1553*)	8.7	−2.9	30-50 μg/kg/d	+ 0.3 (0.8y)	0.8		+ 0.4	−2.6
	12	Male	c.223 T > C (p.Trp75Arg)	5.5	−2.0	30-50 μg/kg/d	+ 0.7	1.0		+ 0.7	−1.3
	13	Female	c.223 T > C (p.Trp75Arg)	8.3	−1.9	30-50 μg/kg/d	+ 0.7	1.0		+ 0.7	−1.2
	14	Male	c.1425delA (p.Val478Serfs*)	7.4	−2.9	30-50 μg/kg/d	+ 0.4	3.0		+ 0.4	−1.7
Present study											
	1	Female	c.7465 T > C (p.Gln2364Pro)	5.6	−2.09	40 μg/kg/d	+ 0.33	3.9	GnRHa for 2 y	+ 0.26	−1.07
	2	Male	c.7465 T > C (p.Gln2364Pro)	7.8	−0.88	50 μg/kg/d	+ 0.27	5.0	GnRHa for 1 y	+ 0.11	−0.31
	3	Male	c.7465 T > C (p.Gln2364Pro)	6.4	−3.74	60 μg/kg/d	+ 0.23	7.7		−0.0	−3.75

*GH* growth hormone, *SDS* standard deviation score, *GnRHa* gonadotropin releasing hormone agonist, *AH* adult height

## Discussion

Human height is a highly heritable trait that involves many genes [22]. In this study, we identified a novel *ACAN* gene pathogenic variant (c.7465 T > C) and found that mean adult height was − 5.2 ± 0.7 SDS and − 3.9 ± 0.9 SDS in male and female variant carriers, respectively. Studies of human mating preferences with respect to height have found that short men prefer small height differences [23]; therefore, in the family studied here, the shortness of both parents might cumulatively affect the heights of their descendants. Our current study of a large family provides the first evidence from a Chinese population that an *ACAN* gene variant can cause low adult height in the absence of a high incidence of familiar bone malformation.

*ACAN* encodes aggrecan, which is the main proteoglycan in the extracellular matrix (ECM) of cartilage [24]. Aggrecan binds chondroitin sulfate and keratan sulfate to its central region, and it forms large aggregates with hyaluronan polymer through its N-terminal globular domain (G1) to form the cartilage ECM scaffold [25]. Aggrecan has two additional globular domains (G2 and G3) that flank its central region. The G3 domain contains a C-type lectin-binding domain that is important in interactions with other extracellular proteins [25]; the function of the G2 domain is unknown. Although aggrecan dysfunction is strongly associated with chondropathy and distinct phenotypes, only a few patients with aggrecan deficiency have been reported, likely due to the wide phenotypic spectrum of *ACAN* mutations [26]. Patients with aggrecan mutations can remain undiagnosed owing to their presentation of clinically less significant conditions, such as ISS [4, 12, 26, 27]. Some studies have identified *ACAN* mutations among patients with syndromic short stature conditions such as spondyloepiphyseal dysplasia, Kimberley type, short stature with early-onset osteoarthritis and/or osteochondritis dissecans [28–30]; short stature might be the most obvious manifestation of *ACAN* mutations [26]. A recent study showed that *ACAN* nonsense mutations are associated with short stature without advanced bone age among Chinese individuals [12]. This evidence and the findings of our study suggest that environmental factors or other undetected genetic variations also influence the manifestations of patients with *ACAN* mutations.

To date, 25 pathogenic *ACAN* mutations have been reported in 24 families with the dominant form of short stature [11, 12, 26]. The reported mutations were present in all domains of the gene; however, all of the mutations disrupted the integrity of at least one of the aggrecan globular domains (G1, G2 or G3) [26]. The association between *ACAN* mutations and adult height suggests that these *ACAN* mutations impair chondrogenesis in a similar way. In mice, aggrecan deficiency follows a recessive pattern, and homozygous deletion of aggrecan is perinatally lethal [31, 32]. Similarly, in humans, the homozygous missense mutation (p. Asp2267Asn) in *ACAN* causes extreme short stature and skeletal dysplasia [30], whereas patients heterozygous for the mutation have less severe phenotypes. It is likely that the impairment of growth plate chondrogenesis is due to insufficient function rather than gain-of-function.

Considering the progressive development of short stature among patients with *ACAN* mutations [25], GH-based treatment is used to rescue height deficiency and prevent further height loss. We found that, among patients with *ACAN* mutations, the response to GH treatment during the first year was generally poor and was correlated with the overall response to GH treatment; however, patients with *ACAN* mutations treated with GH were 5–8 cm taller than their same-sex family members [11]. GH promotes height development by stimulating IGF-1 production and chondrocyte differentiation; therefore, aggrecan deficiencies are unlikely to be repaired by GH alone. Future research to identify molecules to restore normal ECM is needed to improve treatment options for children with *ACAN* mutations.

## Conclusions

We have identified a novel variant in the *ACAN* gene associated with minor bone abnormality without a high incidence of familiar bone malformation. Response to GH therapy was poor compared with the effect in GHD children; however, our results and those of other studies support the view that long-term GH therapy is beneficial for preventing age-accompanied cumulative deterioration of growth loss. Earlier genetic diagnosis and long-term therapy would be useful to obtain better clinical outcomes for children with *ACAN* mutations.

## Additional files

Additional file 1: **Figure S1.** The amino acid "Q" in red represents the conservative property in position "p. Gln2364Pro" among differet species. (PDF 416 kb)

Additional file 2: **Figure S2.** Black arrow: Cervical-vertebral cleft in the spine. White arrows: Apophyses in the upper and lower thoracic vertebrae. (PDF 2309 kb)

## Abbreviations

BA: Bone age
BMI: Body mass index
BWA: Burrows-wheeler aligner
ECM: Extracellular matrix
GH: Growth hormone
GHD: Growth hormone deficiency
HGMD: Human gene mutation database
IGFBP3: IGF-binding protein 3
ISS: Idiopathic short stature
OMIM: Online Mendelian inheritance in man
SDS: Standard deviation score
SGA: Small gestational age

## References

[1] Argente J (2016). Challenges in the Management of Short Stature. Horm Res in Paediatr.

[2] Cohen P, Rogol AD, Deal CL, Saenger P, Reiter EO, Ross JL, Chernausek SD, Savage MO, Wit JM, I.S.S.C.W. participants (2008). Consensus statement on the diagnosis and treatment of children with idiopathic short stature: a summary of the growth hormone research society, the Lawson Wilkins pediatric Endocrine Society, and the European Society for Paediatric Endocrinology Workshop. J Clin Endocrinol Metab.

[3] Grimberg A, DiVall SA, Polychronakos C, Allen DB, Cohen LE, Quintos JB, Rossi WC, Feudtner C, Murad MH (2016). Drug and therapeutics committee and ethics Committee of the Pediatric Endocrine Society. Guidelines for growth hormone and insulin-like growth factor-I treatment in children and adolescents: growth hormone deficiency, idiopathic short stature, and primary insulin-like growth factor-I deficiency. Horm Res Paediatr..

[4] Quintos JB, Guo MH, Dauber A (2015). Idiopathic short stature due to novel heterozygous mutation of the aggrecan gene. J Pediatr Endocrinol Metab.

[5] Shima H, Tanaka T, Kamimaki T, Dateki S, Muroya K, Horikawa R, Adachi M, Naiki Y, Tanaka H, Mabe H (2016). Japanese. Systematic molecular analyses of SHOX in Japanese patients with idiopathic short stature and Leri-Weill dyschondrosteosis. J Hum Genet.

[6] Caliebe J, Broekman S, Boogaard M, Bosch CA, Ruivenkamp CA, Oostdijk W, Kant SG, Binder G, Ranke MB, Wit JM, Losekoot M (2012). IGF1, IGF1R and SHOX mutation analysis in short children born small for gestational age and short children with normal birth size (idiopathic short stature). Horm Res Paediatr.

[7] Khetarpal P, Das S, Panigrahi I, Munshi A (2016). Primordial dwarfism: overview of clinical and genetic aspects. Mol Genet Genomics : MGG.

[8] Alatzoglou KS, Dattani MT (2010). Genetic causes and treatment of isolated growth hormone deficiency-an update. Nat Rev Endocrinol.

[9] Baron J, Savendahl LF, De L, Dauber A, Phillip M, Wit JM, Nilsson O (2015). Short and tall stature: a new paradigm emerges. Nat Rev Endocrinol..

[10] Baron J, Savendahl L, De Luca F, Dauber A, Phillip M, Wit JM, Nilsson O (2014). Short stature, accelerated bone maturation, and early growth cessation due to heterozygous aggrecan mutations. The J Clin Endocrinol Metab.

[11] van der Steen M, Pfundt R, Maas S, Bakker-van Waarde WM, Odink RJ, Hokken-Koelega ACS (2017). *ACAN* gene mutations in short children born SGA and response to growth hormone treatment. J Clin Endocrinol Metab.

[12] Hu X, Gui B, Su J, Li H, Li N, Yu T, Zhang Q, Xu Y, Li G, Chen Y, Qing Y, Li C, Luo J, Fan X, Ding Y, Li J, Wang J, Wang X, Chen S, Shen Y, Chinese Genetic Short Stature, Consortium (2017). Novel pathogenic ACAN variants in non-syndromic short stature patients. Clin Chim Acta.

[13] Li H, Ji CY, Zong XN, Zhang YQ (2009). Height and weight standardized growth charts for Chinese children and adolescents aged 0 to 18 years. Zhonghua Er Ke Za Zhi (Chinese J Pediatr).

[14] Patel L, Clayton PE (2012). Predicting response to growth hormone treatment. Indian J Pediatr.

[15] Wang K, Li M, Hakonarson H (2010). ANNOVAR: functional annotation of genetic variants from high-throughput sequencing data. Nucleic Acids Res.

[16] McLaren W, Pritchard B, Rios D, Chen Y, Flicek P, Cunningham F (2010). Deriving the consequences of genomic variants with the Ensembl API and SNP effect predictor. Bioinformatics.

[17] Kumar P, Henikoff S, Ng PC (2009). Predicting the effects of coding non-synonymous variants on protein function using the SIFT algorithm. Nat Protoc.

[18] Adzhubei IA, Schmidt S, Peshkin L, Ramensky VE, Gerasimova A, Bork P, Kondrashov AS, Sunyaev SR (2010). A method and server for predicting damaging missense mutations. Nat Methods.

[19] Schwarz JM, Rodelsperger C, Schuelke M, Seelow D (2010). MutationTaster evaluates disease-causing potential of sequence alterations. Nat Methods.

[20] Pollard KS, Hubisz MJ, Rosenbloom KR, Siepel A (2010). Detection of nonneutral substitution rates on mammalian phylogenies. Genome Res.

[21] Biasini M, Bienert S, Waterhouse A, Arnold K, Studer G, Schmidt T, Kiefer F, Cassarino TG, Bertoni M, Bordoli L, Schwede T (2014). SWISS-MODEL: modelling protein tertiary and quaternary structure using evolutionary information. Nucleic Acids Res.

[22] Wit JM, Oostdijk W, Losekoot M, van Duyvenvoorde HA, Ruivenkamp CA, Kant SG (2016). Mechanisms in endocrinology: novel genetic causes of short stature. Eur J Endocrinol.

[23] Stulp G, Buunk AP, Pollet TV, Nettle D, Verhulst S (2013). Are human mating preferences with respect to height reflected in actual pairings?. PLoS One.

[24] Sophia Fox AJ, Bedi A, Rodeo SA (2009). The basic science of articular cartilage: structure, composition, and function. Sports health.

[25] Aspberg A (2012). The different roles of aggrecan interaction domains. J Histochem Cytochem.

[26] Gkourogianni A, Andrew M, Tyzinski L, Crocker M, Douglas J, Dunbar N, Fairchild J, Funari MF, Heath KE, Jorge AA (2017). Clinical characterization of patients with autosomal dominant short stature due to Aggrecan mutations. J Clin Endocrinol Metab.

[27] Dateki S, Nakatomi A, Watanabe S, Shimizu H, Inoue Y, Baba H, Yoshiura KI, Moriuchi H (2017). Identification of a novel heterozygous mutation of the Aggrecan gene in a family with idiopathic short stature and multiple intervertebral disc herniation. J Hum Genet.

[28] Gleghorn L, Ramesar R, Beighton P, Wallis G (2005). A mutation in the variable repeat region of the aggrecan gene (AGC1) causes a form of spondyloepiphyseal dysplasia associated with severe, premature osteoarthritis. Am J Hum Genet.

[29] Stattin EL, Wiklund F, Lindblom K, Onnerfjord P, Jonsson BA, Tegner Y, Sasaki T, Struglics A, Lohmander S, Dahl N, Heinegard D, Aspberg A (2010). A missense mutation in the aggrecan C-type lectin domain disrupts extracellular matrix interactions and causes dominant familial osteochondritis dissecans. Am J Hum Genet.

[30] Tompson SW, Merriman B, Funari VA, Fresquet M, Lachman RS, Rimoin DL, Nelson SF, Briggs MD, Cohn DH, Krakow D (2009). A recessive skeletal dysplasia, SEMD aggrecan type, results from a missense mutation affecting the C-type lectin domain of aggrecan. Am J Hum Genet.

[31] Watanabe H, Kimata K, Line S, Strong D, Gao LY, Kozak CA, Yamada Y (1994). Mouse cartilage matrix deficiency (cmd) caused by a 7 bp deletion in the aggrecan gene. Nat Genet.

[32] Lauing KL, Cortes M, Domowicz MS, Henry JG, Baria AT, Schwartz NB (2014). Aggrecan is required for growth plate cytoarchitecture and differentiation. Dev Biol.

