# Supplementary material for: Novel aggrecan variant, p. Gln2364Pro, causes severe familial nonsyndromic adult short stature and poor growth hormone response in Chinese children
Source: BMC Med Genet. 2018 May 16;19:79. doi: 10.1186/s12881-018-0591-z (PMC5956957; doi:10.1186/s12881-018-0591-z)
Supplement: Supplementary file 1 — Figure S1. The amino acid "Q" in red represents the conservative property in position "p. Gln2364Pro" among differet species. (PDF 416 kb) [file 12881_2018_591_MOESM1_ESM.pdf]

Homo sapiens	E Q Q S H L S S I V T P E E Q E F V N N N
Chimp	E Q Q S H L S S I V T P E E Q E F V N N N
Gorilla	E Q Q S H L S S I V T P E E Q E F V N N N
Orangutan	E Q Q S H L S S I V T P E E Q E F V N N N
Gibbon	E Q Q S H L S S I V T P E E Q E F V N N N
Rhesus	E Q Q S H L S S I V T P E E Q E F V N N N
Callithrix jacchus	E Q Q S H L S S I V T P E E Q T L S T E R
Sus scrofa	E Q Q S H L S S I V T P E E Q E F V N N N
Canis lupus familiaris	E Q Q S H L S S I V T P E E Q E F V N N N
Equus caballus	E Q Q S H L S S I V T P E E Q E F V N N N
Ailuropoda melanoleuca	E Q Q A H L S S I V T P E E Q E F V N N N
Pongo abelii	E Q Q S H L S S I V T P E E Q E F V N N N
Nomascus leucogenys	E Q Q S H L S S I V T P E E Q E F V N N N
Macaca mulatta	E Q Q S H L S S I V T P E E Q E F V N N N
Bos Taurus	E Q Q S H L S S I V T P E E Q E F V N N N
