# Supplementary figures and images for: Novel aggrecan variant, p. Gln2364Pro, causes severe familial nonsyndromic adult short stature and poor growth hormone response in Chinese children
Source: BMC Med Genet. 2018 May 16;19:79. doi: 10.1186/s12881-018-0591-z (PMC5956957; doi:10.1186/s12881-018-0591-z)

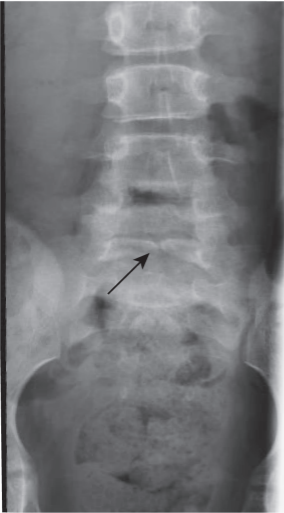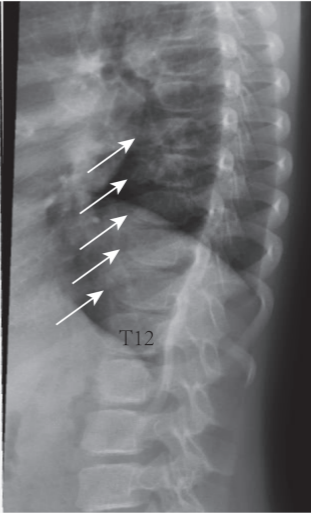

Supplement: Supplementary file 2 — Figure S2. Black arrow: Cervical-vertebral cleft in the spine. White arrows: Apophyses in the upper and lower thoracic vertebrae. (PDF 2309 kb) [file 12881_2018_591_MOESM2_ESM.pdf]
